# Eight principles of neuro-inclusion; an autistic perspective on innovating inclusive research methods

**DOI:** 10.3389/fpsyg.2024.1326536

**Published:** 2024-02-27

**Authors:** Jessica Dark

**Affiliations:** Department of Organizational Psychology, School of Business, Economics and Informatics, Birkbeck, University of London, London, United Kingdom

**Keywords:** autism, autistic researcher, inclusive methods, neurodiversity, participatory research

## Abstract

In this article I explain the value of autistic perspectives in research and argue that support for autistic scholars, community leaders and professionals are required as an inclusive research consideration. I propose consolidation, innovation, and evaluation of inclusive research principles, with consideration given to epistemic agency, autistic participation, and actionable research outcomes. I then present “Eight Principles of Neuro-Inclusion,” a reflexive tool that I have designed as a way of encouraging new developments of inclusive research practices. Through flexible application of this approach, it is hoped that innovative new inclusive methods will materialize, in pursuit of epistemic justice, and in support of actionable research outcomes that benefit our autism community.

## Introduction

Autism is a neuro-developmental condition that is medically characterized in the DSM-5-TR as persistent challenges across all three social criteria; social communication, social interaction, and social reciprocity, in addition to meeting two of the four categories related to restrictive and repetitive behavior, including repetitive activities, restrictive interests, following routines and rituals and repetitive sensory behaviors (American Psychiatric Association [APA], 2013, 2022). However, from a neurodiversity perspective autism is regarded as a natural variation of the human genome (Singer, 2016), and from an embodiment stance; an integral aspect of who a person is (De Jaegher, 2013). There is current debate regarding which language should be used when discussing autism in research (Flowers et al., 2023), with no consensus currently established (Botha et al., 2023). As such, I have chosen to use identity first language; autistic person, rather than person with autism, and I do not capitalize the term “autism” within the writing of this article. I have selected these language choices to affirm my alignment with neurodiversity and embodiment perspectives, thereby recognizing autism as a neuro-type under the umbrella of neurodiversity, with unique strengths and challenges that diverge from prominent ways of thinking and behaving. Due to the continuous development of language associated with neurodiversity, in Table 1 share a glossary of terms used in this article.

**TABLE 1:** A glossary of terms.

Term	Definition
Neurodiversity	Neurodiversity is a concept that recognizes and celebrates the diversity of neurological differences across humanity.
Neuro-inclusion	Neuro-inclusion is a term that refers to creating environments, practices and processes that are inclusive of all people’s cognitive processing requirements. Neuro-inclusion is also the act of listening to and valuing the views and support requirements of people whose thinking and behavior significantly diverge from prominent expressions.
Neuro-profile	A neuro-profile refers to the different cognitive processing traits that as a collective inform a person’s strengths and challenges.
Neuro-trait	A neuro-trait refers to one cognitive function that informs a person’s neuro-profile. For example, executive functions, and differences with short term memory are considered neuro-traits.
Neuro-type	A neuro-type are neuro-traits and behavioral descriptors that have been grouped for diagnostic purposes. For example, autism, ADHD, dyslexia, and dyspraxia are all neuro-types under the umbrella of neurodiversity.

The way that autism is represented in research is currently regarded as an epistemic injustice (Catala et al., 2021). The literature is awash with stereotypes (Draaisma, 2009) and derogatory language (Botha and Cage, 2022) that shape how autism is understood (Botha et al., 2023). Emancipatory research has informed different ways of working with disability groups, including the use of inclusive research methods and participatory approaches. These methods aim to include disabled participants in research either through adapting research materials and procedures (Aldridge, 2012; Ellard-Gray et al., 2015), or by actively engaging participants as co-researchers throughout the research process (Aldridge, 2012; 2016; Brown, 2021).

In this article I discuss why consolidation, evaluation, and innovation of inclusive research principles are required for improved inclusion of autistic people in research, and argue why inclusion of autistic scholars, community leaders, and professionals should be considered under the umbrella of inclusive research methods. I then present “Eight Principles of Neuro-Inclusion” a reflexive tool that I have designed as a way of encouraging uptake and development of inclusive research methods, in support of better understanding and improved life opportunities for our autism community.

## Why new approaches are needed for the inclusion of autistic people in research?

Autistic people currently experience many inequalities across society. COVID-19 informed glaring inequalities for autistic people accessing healthcare (Oakley et al., 2021). Health inequalities have also been demonstrated by some autistic groups being unable to access a timely diagnosis (Leedham et al., 2019). Education is also a concern, with many autistic children unable to access appropriate schooling (Hasson et al., 2022). Furthermore, the low employment figure for autistic people at 22% (The Autism Employment Gap, 2021) demonstrates that our autism community are experiencing exclusion across the entirety of their lifespan, and experience inequality across all aspects of society. Amidst these challenging times, autistic people have raised concern that research does not reflect their personal understanding of autism (Gowen et al., 2020). If actionable research outcomes that increase the understanding of autism are to materialize, how research is conducted must be considered in relation to inclusive research methods, in order to improve the impact of the research produced.

Inclusive and participatory approaches are valuable research methods in support of marginalized communities (Aldridge, 2012). However, current approaches are not without challenges. For instance, it is recognized that when conducting participatory research there may be challenges with funding and the coordination and allocation of roles (König et al., 2022). Furthermore, the research process may require additional implementation time when compared with traditional methods (Hewitt et al., 2023). Despite an inclusive approach increasing community engagement (Aldridge, 2012), challenges with demographic recruitment, and autistic representation in research, are not frequently addressed. As such, varied and innovative approaches to inclusive methods are required for an improved understanding of autism to materialize.

## The autistic researcher

It is often assumed that the researcher voice is that of non-autistic scholars, and for the large part this is true. However, it is recognized that the incorporation of autistic voices leads to higher quality research (Bertilsdotter Rosqvist et al., 2019) and that autistic researchers bring unique strengths to the interpretation of data (Grant and Kara, 2021). Academia is regarded a good career path for autistic people who are pursuing research topics that they are passionate about (Jones, 2023). As such, there is a growing number of autistic people studying and working in academia that conduct research with their own autism community (Bertilsdotter Rosqvist et al., 2023; Fletcher-Watson et al., 2021). Furthermore, there are many autistic community members and professionals, that if appropriately supported to lead practitioner research, would inform the phenomena in uniquely meaningful ways.

Encouraging autistic people to lead research surpasses diagnostic labeling alone. Autistic scholars bring specific skills to research, such as attending to topics of interest for prolonged periods and paying good attention to detail (Grant and Kara, 2021). The lived experience of the autistic researcher informs a level of shared-neuro-trait relevancy and empathy to the interpretation of data (Grant and Kara, 2021). Thus, autistic researchers are able to draw from both personal insights and extensive study in the field, or broad clinical and community involvement, rather than relying on literature alone to inform their understanding of the phenomena.

Despite the benefits of autistic led research; academia poses its own challenges. It is noted that as a profession, there is a tendency to overwork (Jones, 2023), with a “dutiful” mentality that encourages researchers to “get on with things, even when it causes them burnout or distress” (Bertilsdotter Rosqvist et al., 2023, p.1236). Furthermore, the environmental demands of studying and challenges associated with appropriate understanding and support at university (Gurbuz et al., 2019) likely act as a barrier or deterrent to those considering a research career. As such, I propose that for autistic scholars, community leaders and professionals to successfully undertake research with their own community, consideration of all autistic people’s support needs must be recognized under the umbrella of inclusive research methods.

## Eight principles of neuro-inclusion

In support of improved inclusion of autistic people in research I have developed the “Eight Principles of Neuro-Inclusion” outlined in Table 2, a reflexive tool that can be used by scholars of all neuro-types when working with autistic participant groups to inform their inclusive research practice. The approach is considerate of method, procedure, and dissemination of research, whilst also being mindful of both the autistic participant and the autistic scholars support needs. I hope that as further developments in the field of inclusive research methods progress, broader autistic demographics and hard-to-reach community views will be included in research, in addition to encouraging and supporting more autistic practitioners and scholars to lead, facilitate, and disseminate research with their own autism community. The following sub-headings present each principle in turn, explaining how they inform inclusive considerations when conducting research with autistic demographic groups.

**TABLE 2:** Eight principles of neuro-inclusion.

Principle	Explanation
Respect	Respect the autonomy and perceptual positionality of all autistic people.
Representation	Include under-researched participant groups such as non-verbal communicators, women, non-binary, trans gender, and people of color. For broader demographic groups include intersectional considerations so that accumulative challenges can be reflected upon in relation to the phenomena.
Learn and Experiment	Learn from the wealth of information already available on inclusive research methods and consider an experimental approach to applying traditional methods in inclusive ways.
Remove Barriers	Evaluate the study design by reflecting upon personal, social, and societal barriers to participation and how these can be removed or minimized to accommodate autistic participants and autistic researchers.
Inclusion of Participants	As per participatory principles, consider the inclusion of participants throughout the research process. Define roles and responsibilities and evaluate researcher and participant power-dynamics.
Actionable Outcomes	Inform actionable research outcomes by considering the context, culture, and demographic of research, with an aim to assist comparison of data and application of findings to real-life contexts.
Language Choices	Contemplate what language will be used in the recruitment, write-up and dissemination of research and the messages that words and terms portray about autistic people.
Dissemination	Create a dissemination plan that is considerate of how autism is discussed and in a format that is accessible to the participant demographic who informed the research.

### Principle 1-respect

The foundation of inclusive research practices must be “respect” – “Researchers need to actively demonstrate that they value lived experience” (Nicolaidis et al., 2019, p.2012). Respecting the unique and varied expressions of autism, as informed by autistic people, is paramount when conducting research with autistic participant groups (Fletcher-Watson et al., 2019). The principle for respect is borne out of the recognition that autistic people’s experiences and perception of the world is an embodiment of self (De Jaegher, 2013) with thinking, behavior and experiences of the world uniquely informed by people’s underlying autistic neurology (Sinclair, 1993; Cooper et al., 2020). Therefore, to understand autistic people’s perceptions of the world, first recognition of autistic perceptual differences (Milton, 2012) is required.

As advised by non-autistic scholar Thompson-Hodgetts (2022), researcher positionality is an important reflexive consideration for all scholars conducting research alongside autistic participants. A recent survey by Botha and Cage (2022) included the views of 195 autism researchers who were asked five open-ended questions related to autism research. Results indicated that greater inclusion of autistic people reduced ableist cues in the discussion of autism, thereby evidencing how a community informed approach, not only promotes autistic voices to be heard, but also researchers who adopt this way of working, better align their thinking with a community understanding. As such, informing research from a community perspective is beneficial to autistic people’s representation in research regardless of the neuro-type of the researcher. Therefore, I suggest that adopting an autism community informed approach is a respectful way to conduct research with autistic people that all researchers of all neuro-types can implement.

### Principle 2-representation

Research often includes a WEIRD; White, Educated, Industrialized, Rich, Democratic participant sample (Henrich et al., 2010). Participant homogeneity can be beneficial when gaining consensus within a group, but it has limitations when understanding diversity across a demographic (Apfelbaum et al., 2014). Inclusive research often aims to include a range of different autistic people across the broad scope of the autism spectrum; albeit not all at once. As such, active recruitment of under-represented autistic groups, such as non-verbal communicators (Hickman, 2022), women (D’Mello et al., 2022), non-binary, transgender (Gratton et al., 2023), and people of color (Malone et al., 2022) should be considered. Furthermore, for broad demographic samples it may be beneficial to evaluate intersectional differences, such as gender, race, and socio-economic status, so that accumulative challenges can be understood across the participant sample, in relation to the phenomena (Cascio et al., 2020).

### Principle 3-learn and experiment

Principle three suggests that an experimental approach to applying traditional research methods is required for the inclusion of autistic people. Traditional research methods have a wealth of reliability and validity supporting their use. However, application of traditional techniques is often underpinned by rigid and directive guidance that may not be suitable for autistic researchers to implement, nor does it support effective engagement of autistic participants in research.

Activities associated with learning from and adapting traditional methods could include, reviewing guides on inclusive research practice, and drawing from articles that use or evaluate inclusive and participatory approaches. Adapting methods involves considering alternative ways of applying traditional techniques, such as a walk and talk interview style to aide participant engagement (North, 2021), or using creative methods to support participants to express their views in alternative ways (Lewis et al., 2023). Through learning from, evaluating, and applying traditional methods in new ways, the collective understanding of inclusive research will improve, and over time new inclusive methods will become proven ways of working in their own right.

### Principle 4-remove barriers

Different autistic participant groups may require different approaches to inclusion (Fletcher-Watson et al., 2019). For instance, an autistic participant sample with low language levels or who are in assisted employment, may require a different approach to an autistic participant group who are living and working independently. Thus, it is beneficial to reflect upon how the chosen method impacts the participant (Nicolaidis et al., 2019). For instance, it may be beneficial to provide an online interview to mitigate the cognitive demands of traveling to an in-person location (Crane et al., 2023) or provide rest breaks during an interview (North, 2021) to reduce the impact of social and sensory demands.

It is also beneficial to evaluate and remove barriers for the autistic researcher by considering what adjustments are required when leading research. For instance, it is evidenced that same neuro-type communication is more effective than across different neuro-type communication (Crompton et al., 2020; Davis and Crompton, 2021). As such, discussion of working arrangements that are considerate of autistic neuro-type requirements should be reflected upon prior to commencing research for mixed neuro-type teams (Bertilsdotter Rosqvist et al., 2023).

Accommodations may surpass the social aspects of research and relate to research activities. For instance, I personally experience auditory and language processing challenges and eye-tracking difficulties that makes transcribing interviews by hand challenging. Therefore, I require assistance from a transcription service to mitigate these difficulties. In recognition of the variation of support required among autistic people, each autistic researchers support needs will be personal to them and should be accommodated accordingly.

### Principle 5-inclusion of participants

As per participatory principles, involving the community who are subject of research, elevates their collective voice to a position of authority on their own experiences (Aldridge, 2016), and increases the relevancy and impact of the research (Kaplan-Kahn and Caplan, 2023). The level of community participation will depend on the approach adopted and study design; however, scholars may find reference to Aldridge’s “Participatory Model,” a useful tool when considering different levels of participation (Aldridge, 2016. p. 156). However, it should also be noted that participation boundaries may not be as clearly defined in research, as they are on paper, and as such it may be helpful to consider participation as a continuum (Brown, 2021). Thus, regardless as to whether a full or partial participatory approach is adopted, it is beneficial to define and communicate responsibilities for all involved (Aldridge, 2016) including external partner roles (Nicolaidis et al., 2019). Further participatory considerations could include, reflecting on the role of data ownership and authorship (Wallis and Borgman, 2012) and evaluating power-dynamics between the researcher and participant (Aldridge, 2016; Nicolaidis et al., 2019) with consideration of how these dynamics inform the co-created research.

### Principle 6-actionable outcomes

Research outcomes that inform the understanding, supports and life opportunities of autistic people should be central to an inclusive research design. In support of actionable research outcomes, I propose that the data collected with autistic participants is culturally and contextually appropriate (Barnes, 2004) and that the demographic criteria informing the research are explicitly stated (Fletcher-Watson et al., 2021). As emphasized in the introduction, autistic people face challenges across all aspects of society, thus research is required to progress understanding, inform support requirements, and improve the life opportunities for this demographic. Through consideration of specific contexts and demographic features, research outcomes can be easily compared across multiple articles and findings considered in relation to real-life contexts (Pellicano et al., 2014) thereby, improving the understanding of established literature as a means to identify research gaps and build on existing knowledge.

### Principle 7-language choices

The choice of language used in research is an important consideration (Monk et al., 2022). Language has connotations that inform a specific way of thinking about autistic people (Bottema-Beutel et al., 2021) and language associated with neurodiversity is very different to that of pathologizing medical terms (Walker, 2021). Thus, a reflexive approach to what language is used is beneficial. Evaluation of language could include reflecting on whether the language used, and messages portrayed, could be deemed “potentially offensive” to autistic people (Monk et al., 2022, p.791), and taking the time required to remove ableist and dated descriptors (Bottema-Beutel et al., 2021). In addition to defining and consistently applying terms when adopting a neurodiversity perspective, thus assisting comprehension due to the developing nature of the associated language (Walker, 2021).

### Principle 8-dissemination

The final principle to be discussed is dissemination of research. How findings are shared after the research has been published is an important reflexive consideration. It is beneficial to consider how research findings are accessible by the community who inform the study. As such, the demographic profile of the community should also inform how the findings are disseminated, with consideration given to minimizing stigma and harm (Nicolaidis et al., 2019). Dissemination of research can be achieved through varied and creative approaches. Ross-Hellauer et al. (2020) shares helpful considerations when planning dissemination of research, including but not limited to, reflecting on the aims and objectives of dissemination, promoting visibility of work, and considering creative formats, such as lay-summaries, blogs, diagrams, and talks.

## Discussion

This paper recognizes that for epistemic progress to be made, the consolidation, innovation, and evaluation of current inclusive and participatory research approaches are required. Furthermore, how autistic researchers, community leaders and professionals are supported to lead research should be added to the range of inclusive considerations explored under this phenomenon. The “Eight Principles of Neuro-Inclusion” is presented in this paper as a tool to inform researcher reflexivity when conducting research with autistic communities. However, the principles are not directive and can be flexibly applied. As such, the same principles could be drawn upon to inform inclusive research with different neuro-types and more broadly across different disability groups.

In application of the reflexive approach presented in this paper, I hope that there will be a greater uptake and exploration of new inclusive ways of researching autistic communities. I conclude this commentary with a call for action. I believe that it will take a community effort for inclusive approaches to become mainstream consideration. However, if more scholars consolidate, innovate, and evaluate their approach to research, epistemic justice through appropriate representation of autistic people in research will materialize, leading to better life opportunities for our autism community across society.

## Data availability statement

The original contributions presented in this study are included in this article/supplementary material, further inquiries can be directed to the corresponding author.

## Author contributions

JD: Conceptualization, Writing−original draft, Writing–review and editing.
